# Variation in Salivary Parameters and its Correlation with Plaque and Gingival Status among 12 to 15 Years Schoolchildren of Rural and Urban Jaipur City in Winter and Summer Seasons

**DOI:** 10.5005/jp-journals-10005-1132

**Published:** 2012-02-24

**Authors:** Anupama Gaur, N Anup, Rajesh Sharma

**Affiliations:** Senior Lecturer, Department of Public Health Dentistry, Jaipur Dental College, A/33, Van Vihar Colony, Opposite Kamal and Company Tonk Road, Jaipur-302017, Rajasthan, India, e-mail: anupama.sharma11@yahoo.com; Professor and Head, Department of Public Health Dentistry, Jaipur Dental College, Jaipur, Rajasthan, India; Associate Professor, Department of Pedodontics and Preventive Dentistry, Jaipur Dental College, Jaipur, Rajasthan, India

**Keywords:** Seasonal variations, Unstimulated and stimulated salivary parameters, GC saliva check buffer kit, Plaque and gingival index, Schoolchildren

## Abstract

**Background:** Saliva circulating in the mouth at any given time is termed as whole saliva and comprises of mixtures of secretions from major and minor salivary glands and traces from gingival crevicular fluid. This saliva in the form of unstimulated/stimulated salivary parameters governs the overall homeostatic mechanism of mouth.

**Aim:** To evaluate variation in salivary parameters and its correlation with plaque and gingival status among 12 to 15 years schoolchildren of rural and urban Jaipur city in winter and summer season.

**Objectives:** To evaluate unstimulated/stimulated salivary parameters amongst 12 to 15 years schoolchildren of rural and urban Jaipur city in winter and summer using GC saliva check buffer kit by GC America Inc. To correlate unstimulated/ stimulated salivary parameters with plaque and gingival status among 12 to 15 years schoolchildren of rural and urban Jaipur city in winters and summer season.

**Methodology:** Eighty schoolchildren of age group between 12 and 15 years were included in this study. They were divided into two groups, group 1—comprised of schoolchildren belonging to rural area and group 2—comprised of schoolchildren belonging to urban area. Each group was further divided into government and private schoolchildren, comprising of 10 subjects in each of the two schools.

Study participants underwent clinical examination, and examination of salivary samples for qualitative and quantitative analysis of unstimulated/stimulated salivary parameters in winter and summer season.

**Results:** No difference in resting salivary flow rate was observed between the two seasons but was found to be comparatively higher among urban schoolchildren as compared to rural. Viscosity of saliva increases in winter as compared to summer among rural schoolchildren. Saliva quantity was found to be very low in summer as compared to winter among rural schoolchildren. Salivary buffering capacity was found to be lower in winter season irrespective of the difference in area. Salivary pH scores were found to be higher in summer as compared to winter among both rural and urban schoolchildren, and this difference was statistically significant (<0.05). Mean plaque scores were found to be higher in winter as compared to summer. Positive correlation (+0.063) was observed between pH and plaque scores in winter season. Positive correlation (+0.045) was observed between pH and gingival scores in winter season.

**Conclusion:** There is a need for dietary counseling and basic oral health care in the study area irrespective of the season. Food consumption patterns differ significantly in winter as compared to summer thereby affecting the oral clearance rate which directly or indirectly affects unstimulated and stimulated salivary patterns and plaque and gingival status.

**How to cite this article:** Gaur A, Anup N, Sharma R. Variation in Salivary Parameters and its Correlation with Plaque and Gingival Status among 12 to 15 Years Schoolchildren of Rural and Urban Jaipur City in Winter and Summer Seasons. Int J Clin Pediatr Dent 2012;5(1):39-48.

## INTRODUCTION

At the moment an infant takes its first breath of life, a residential microbial community begins to form on the tongue and the oral mucous membranes. Later, with the eruption of the teeth, additional microorganisms immigrate and establish colonies on the sheltering tooth surfaces. As the time passes and the crevice deepens between the teeth and the gingiva, still another flora takes root in this protected niche. The bacteria with the advantage of several billion years of evolution, use every topographical, physical, and metabolic opportunity the mouth provides to maintain their presence. Learning the mechanism involved in bacterial colonization and plaque maturation, and characterizing them at a molecular level is a productive research area for oral ecologists. Equally interesting is the latest research in the counterforce–the host response.^1^

Long before there were toothbrushes, floss and oral water irrigators, the evolutionary process created protective mechanisms in the mouth for self defense and self sustenance against the hundreds of species of microorganisms and their 50 billion (or more) descendants that make their home in their mouth. The major responsibility for the defense of the mouth rests with the salivary glands, the original slow release device fashioned by an ancient technology.^2^

Thus, saliva circulating in the mouth at any given time is termed as whole saliva and it comprises of a mixture of secretions from the major and minor salivary glands and traces from the gingival crevicular fluid. Saliva definitely promotes oral health and hence lack of its secretion contributes to the disease process. The saliva by constantly bathing the teeth and oral mucosa, functions as a cleansing solution, a lubricant, a buffer and ion reservoir of calcium and phosphate which are essential for remineralization of initial carious lesions.^3,4^

Thus, saliva in the form of its unstimulated and stimulated salivary parameters governs the overall oral homeostatic mechanism of the mouth. These parameters vary individually are found to exhibit circadial rhythms in humans due to variation in oral temperature.^5^

Most dentists are concerned that their patients are consuming a record number of sugar-filled sodas, sweetened fruit drinks, and such other stuff that affect their oral environment. Children are invariably the victims of these foodstuffs. These items generally have very little nutritional value, albeit their commercial value. They can take a heavy toll on our teeth over time.^6^ Thus, dietary habits and the choice of food among children and teens are important factors that determine how quickly they may develop oral diseases. Five dental and oral environmental factors, namely, the tooth chemistry, the amount of salivary flow, the types of dental plaque bacteria, the type of fermentable carbohydrate eaten, and the frequency of daily food intake, especially the between meal snacks, are the causative agents concerned with the initiation and extension of dental diseases.^7^

The extent and significance of these interactions in the mouth of people consuming their normal conventional diet possibly affecting the salivary parameters which necessitate a need for this study.

Thus, to design good intervention programs and preventive strategies, information on food habits and dietary intake of the target population is very important.^8^ As Rajasthan is the second largest state of India territorially, encompassing its economy which is primarily agricultural and pastoral. The food in Rajasthan is a diverse as the state itself. In some areas, it is simple and basic while in others it is exotic and elaborate. As it normally happens, the cooking is influenced by the land, lifestyle of its inhabitants, and the avaibility of ingredients in the region. Thus, contributing food patterns comprises mainly of plant origin, which in turn are largely seasonal dependent, thereby affecting the food avaiblity and dietary patterns.^9^ In additionally, food habits also changes due to extreme temperature changes ranging from 7 to 45°C. According to ayurveda, with the changes in season our body needs different foods to adjust itself to the outside environment. In winters along with consumption of routine fruits and vegetables, sweets cuisines containing jaggary/gur are highly increased as they are believed to have warming properties. Similarly in summer seasons along with routine fruits and vegetables consumption of lassi (sweetened yogurt) and chaach (buttermilk) are increased as these are believed to possess excellent coolant properties.^10^ Sweets made out of jaggary/ gur are mainly consumed during winter season and are solid and retentive in nature thereby decreases the oral clearance time, thus affecting the oral environment.

Studies^11,12^ have suggested that differences in the composition of saliva in rural and urban groups are closely associated with differences in absolute and relative amounts of nutrients that with energy content of diet. The high buffer effects of saliva appears to be associated with the exceptionally grain fiber-rich diet. Therefore, variation in the seasons affects the food availability and consumption patterns which in turn may have possible effect on salivary parameters.

## AIM

To evaluate variation in salivary parameters and its correlation with plaque and gingival status among 12 to 15 years schoolchildren of rural and urban Jaipur city in winter and summer season.

## OBJECTIVES

- To evaluate unstimulated/stimulated salivary parameters amongst 12 to 15 years schoolchildren of rural and urban Jaipur city in winter and summer using GC saliva check buffer kit by GC America Inc.
- To correlate unstimulated/stimulated salivary parameters with plaque and gingival status among 12 to 15 years schoolchildren of rural and urban Jaipur city in winters and summer season.

## SUBJECTS AND METHODS

*Study design: *A comparative longitudinal study conducted to evaluate and correlate unstimulated/stimulated salivary parameters with plaque and gingival status amongst 12 to 15 years schoolchildren of rural and urban Jaipur in two different seasons.

## METHODOLOGY

Eighty schoolchildren (40 in winter and 40 in summer) of age group between 12 and 15 years were included in this study. They were divided into two groups: Group 1 – comprised of schoolchildren belonging to rural area and group 2–comprised of schoolchildren belonging to urban area. Each group was further divided into government and private schoolchildren, comprising of 10 subjects in each of the two schools.

Study participants underwent clinical examination, and examination of salivary samples for qualitative and quantitative analysis of unstimulated/stimulated salivary parameters in winter and summer season. Under clinical examination, recording of plaque index (Silness and Loe in 1964)^13^ and gingival index (Loe and Silness in 1963)^14^ was performed on the study subjects. Assessment of unstimulated and stimulated salivary parameters and their status was recorded. General information of the schoolchildren regarding their name, age, sex, class and school name was also collected.

*Ethical clearance: *Ethical clearance was taken from the institutional ethics committee and voluntary informed consent was obtained from the director of the orphanage. Permission to conduct the study was obtained from department of health and family welfare.

*Study procedure: *The study had been carried out from January 2010 to May 2010 in Jaipur district, the capital of Rajasthan. Jaipur district is divided into 13 tehsils, and covers an area of 11117.8 square km and a population of 5,251,071, with rural population of 2,659,004 and urban 2,592,067 and a population density of 471 persons per square Km.^15^ Based on simple random number table, Bhanpur (rural) and Central Jaipur (urban) was selected for the present study. Climate of Rajasthan can be broadly divided into four seasons: Premonsoon/summer, monsoon, postmonsoon and winter. In Jaipur city, maximum average temperature in summer ranges between 33 and 45°C. whereas, minimum temperature ranges between 24 and 27°C. Similarly, in winter maximum average temperature ranges between 20 and 24°C, and minimum temperature ranges between 7 and 10°C.^10^

The list of the schools was collected from the Department of District Education Office, Jaipur (DDEO, Jaipur) and Shiksha Sankul, Jaipur. Prior to conducting the study, the investigator was calibrated at the department of public health dentistry and kappa value was found to be 0.85 which was satisfactory.

## INCLUSION CRITERIA

1. Schoolchildren in the age group of 12 to 15 years.
2. Those who gave the consent.
3. Schoolchildren who were continuous residents since birth were selected (continuous life residents are those who were born, reared and living in the same area).
4. The schoolchildren who were present on the scheduled date of investigation were included.

## EXCLUSION CRITERIA

1. Those who did not gave the consent.
2. Those subjects with findings of any acute oral infections.
3. Subjects with orthodontic appliances.
4. Subjects under antibiotic therapy in the past 6 months.
5. Subjects who underwent oral prophylaxis in last 24 months.

### Armamentarium

Sufficient numbers of sterile instruments were made available for the oral examination during the study.

1. Plane mouth mirrors.
2. Tweezers.
3. Blunt Probe.
4. Explorers.
5. Kidney trays.
6. Containers (one used for instruments and other for sterilized instruments).
7. 10% Korsolex.
8. Sterilized gauze piece and cotton.
9. Disposable gloves and mouth mask.
10. Data recording proforma (salivary parameters and index recording proforma).
11. GC saliva check buffer kit.

### Statistical Analysis

The statistical package for socioscience (SPSS), version 10 was used for analysis. The Chi-square test was used to compare proportion and one-way analysis of variance method was used to compare means. Least square distance multiple comparison tests were used to detect the significant difference between the group’s means.

## RESULTS

### Comparison of Level of Hydration according to Season and Area (Table 1 and Fig. 1)

Out of the total 40 study participants in rural area, 15 (75%) were found to have normal resting flow in winter and summer; whereas, 5 (25%) were found to have low resting flow in both the seasons. Similarly out of the total 40 study participants belonging to urban area, 16 (80%) were found to have normal resting flow in both the seasons and 4 (20%) were found to have low resting flow.

### Comparison of Saliva Consistency according to Season and Area (Table 2 and Fig. 2)

Out of the total participants in rural area 17 (85%) were found to have increased salivary viscosity in summer as compared to winter 5 (25%). Similarly among the urban participants 10 (50%) were found to have increased viscosity in summer as compared to winter 9 (45%).

**Fig. 1:**
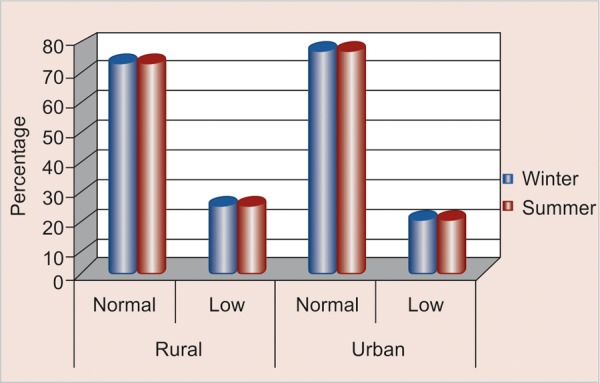
Distribution of level of hydration according to season and area

**Fig. 2:**
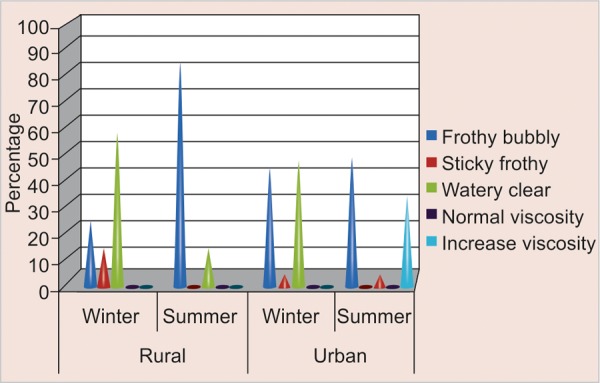
Distribution of saliva consistency according to season and area

### Comparison of Saliva Quantity according to Season and Area (Table 3 and Fig. 3)

Among the study participants in rural area saliva quantity was found to be very low in summer 12 (60%) as compared to winter 1 (5%). Similarly in urban area saliva quantity was found to be very low in summer 5 (25%) as compared to winter 4 (20%); whereas saliva quantity was found to be normal in winters as compared to summer among rural 17 (85%) and urban 10 (50%) participants.

### Comparison of Buffering Capacity according to Season and Area (Table 4 and Fig. 4)

Out of the total study participants belonging to rural area, 3 (15%) were found to have very low buffering capacity in winter as compared to summer, 15 (75%) were found to have low buffering capacity in summer as compared to winter and 7 (35%) were found to have normal buffering capacity in winter as compared to summer; whereas in urban area 2 (10%) were found to have very low buffering capacity in winter as compared to summer, 15 (75%) were found to have low buffering capacity in summer as compared to winter and 10 (50%) were found to have normal buffering capacity in winter as compared to summer.

### Mean pH Scores according to Season and Area (Table 5 and Fig. 5)

The pH scores were found to be higher in winter season (7.47 ± 0.44) as compared to summer (7.18 ± 0.48) among rural and urban participants, but the difference is found to be statistically significant among rural participants (<0.05).

### Mean Plaque Scores (PI) according to Season and Area (Table 6 and Fig. 6)

Plaque scores were found to be higher in summer season as compared to winter among both rural and urban study participants and this difference was found to be not statistically significant (>0.05).

**Table 1:** **Table 1: **Distribution of level of hydration according to season and area

*Level of hydration*		*Rural*				*Urban*			
		*Winter*		*Summer*		*Winter*		*Summer*	
Normal		15 (75.00)		15 (75.00)		16 (80.00)		16 (80.00)	
Low		5 (25.00)		5 (25.00)		4 (20.00)		4 (20.00)	
Total		20 (100.00)		20 (100.00)		20 (100.00)		20 (100.00)	

**Table 2:** **Table 2: **Distribution of saliva consistency according to season and area

*Saliva consistency*		*Rural*				*Urban*			
		*Winter*		*Summer*		*Winter*		*Summer*	
Frothy bubbly		5 (25.00)		17 (85.00)		9 (45.00)		10 (50.00)	
Sticky frothy		3 (15.00)		0 (0.00)		1 (5.00)		0 (0.00)	
Watery clear		12 (60.00)		3 (15.00)		10 (50.00)		1 (5.00)	
Normal viscosity		0 (0.00)		0 (0.00)		0 (0.00)		0 (0.00)	
Increase viscosity		0 (0.00)		0 (0.00)		0 (0.00)		7 (35.00)	
Total		20 (100.00)		20 (100.00)		20 (100.00)		20 (100.00)	

**Table 3:** **Table 3: **Distribution of saliva quantity according to season and area

*Saliva quantity*		*Rural*				*Urban*			
		*Winter*		*Summer*		*Winter*		*Summer*	
Very low		1 (5.00)		12 (60.00)		4 (20.00)		5 (25.00)	
Low		2 (10.00)		6 (30.00)		6 (30.00)		6 (30.00)	
Normal		17 (85.00)		2 (10.00)		10 (50.00)		9 (45.00)	
Total		20 (100.00)		20 (100.00)		20 (100.00)		20 (100.00)	

**Table 4:** **Table 4: **Distribution of buffering capacity according to season and area

*Buffering capacity*		*Rural*				*Urban*			
		*Winter*		*Summer*		*Winter*		*Summer*	
Very low		3 (15.00)		0 (0.00)		2 (10.00)		0 (0.00)	
Low		10 (50.00)		15 (75.00)		8 (40.00)		15 (75.00)	
Normal		7 (35.00)		5 (25.00)		10 (50.00)		5 (25.00)	
Total		20 (100.00)		20 (100.00)		20 (100.00)		20 (100.00)	

**Fig. 3:**
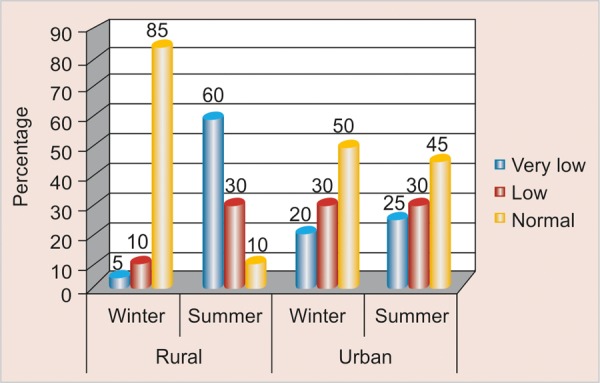
Distribution of saliva quantity according to season and area

**Fig. 4:**
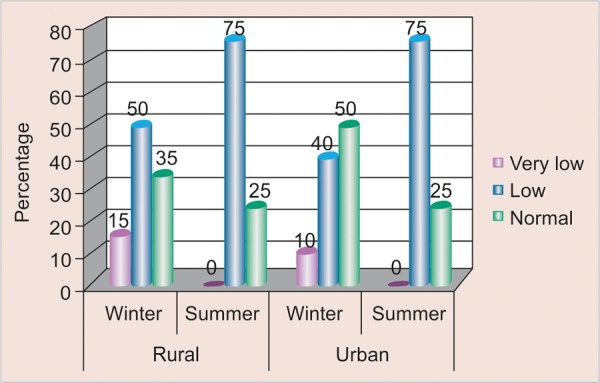
Distribution of buffering capacity according to season and area

**Table 5:** **Table 5: **Mean ± SD of pH according to season among study participants belonging to rural and urban area

*Area*		*Mean ± SD*				*p-value*		*Significance*	
		*Winter*		*Summer*					
Rural		7.47 ± 0.44		7.18 ± 0.48		<0.05		S	
Urban		7.23 ± 0.46		7.09 ± 0.38		>0.05		NS	

S: Significance; NS: Not significant

**Fig. 5:**
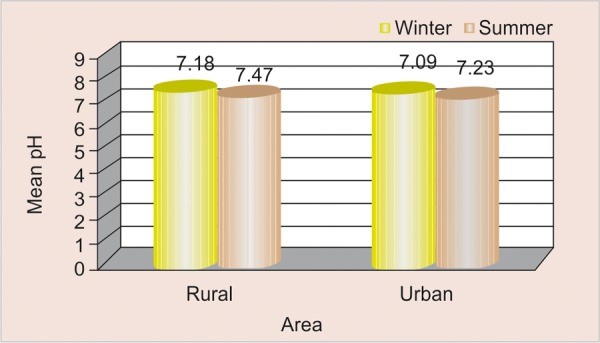
Mean pH scores according to season and area

**Table 6:** **Table 6: **Mean ± SD of plaque index according to season among study participants belonging to rural and urban area

*Area*		*Mean ± SD*				*p-value*		*Significance*	
		*Winter*		*Summer*					
Rural		4.74 ± 2.41		5.11 ± 3.11		>0.05		NS	
Urban		2.50 ± 2.89		3.39 ± 1.88		>0.05		NS	

NS: Not significant

**Fig. 6:**
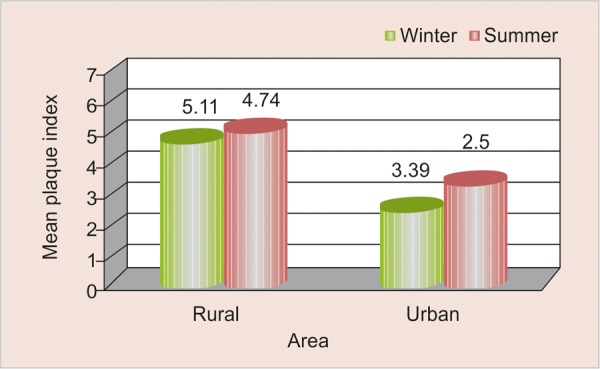
Mean plaque scores according to season and area

### Mean Gingival Scores (GI) according to Season and Area (Table 7 and Fig. 7)

Gingival scores were found to be higher in summer season as compared to winter among both rural and urban study participants and this observed difference was found to be not statistically significant (>0.05).

**Table 7:** **Table 7: **Mean ± SD of gingival index according to season among study participants belonging to rural and urban area

*Area*		*Mean ± SD*				*p-value*		*Significance*	
		*Winter*		*Summer*					
Rural		3.23 ± 3.12		4.89 ± 3.08		>0.05		NS	
Urban		1.70 ± 2.99		2.21 ± 3.09		>0.05		NS	

NS: Not significant

**Fig. 7:**
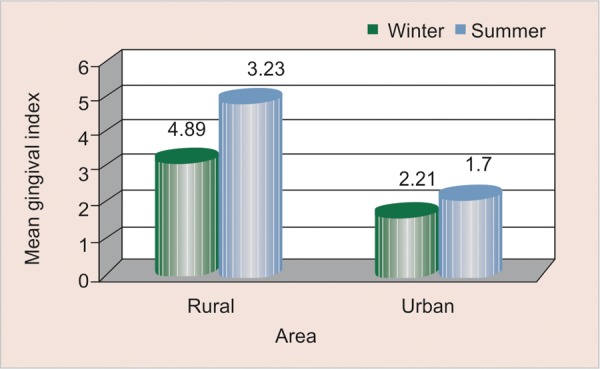
Mean gingival scores according to season and area

### Correlation between pH and Plaque Scores (PI) according to Season amongst Study Participants in Rural Area (Table 8 and Fig. 8)

Among the study participants in rural area negative correlation was observed between pH and plaque scores in winter season (–0.093) and a positive correlation was observed for the same in summer (+0.063), but this difference was found to be not statistically significant (>0.05).

### Correlation between pH and Plaque Scores (PI) according to Season amongst Study Participants in Urban Area (Table 9 and Fig. 9)

Among the study participants in urban area a negative correlation was observed between pH and plaque scores in winter season (–0.115) which was found to be not statistically significant (>0.05). Whereas in summers negative correlation was observed for the same (–0.447) which was found to be statistically significant (<0.05).

**Table 8:** **Table 8: **Correlation between plaque index and pH according to season among study participants belonging to rural area

*Season*		*r-value*		*p-value*		*Significance*	
Winter		–0.093		>0.05		NS	
Summer		+0.063		>0.05		NS	

NS: Not significant

**Fig. 8:**
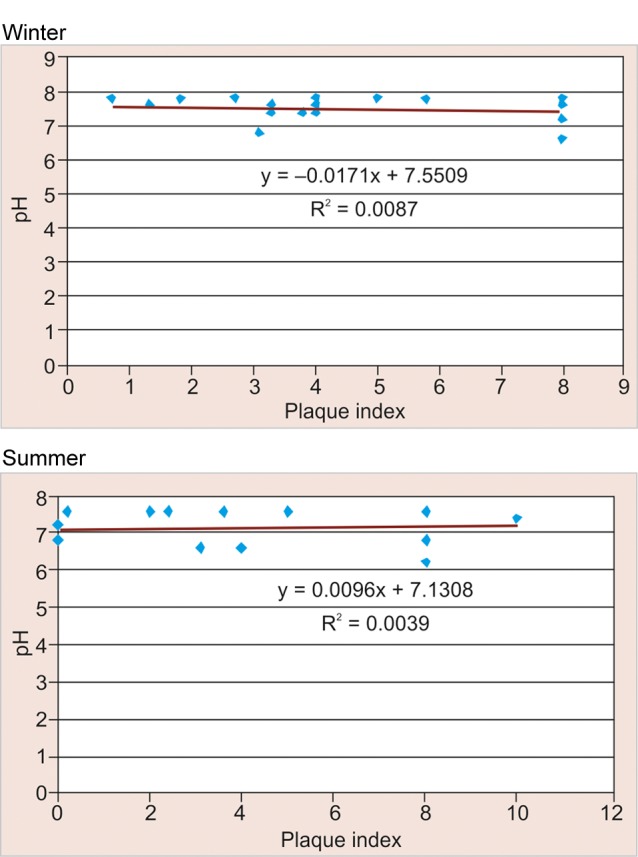
Correlation between pH and plaque scores according to season among study participants in rural area

### Correlation between pH and Gingival Scores (GI) according to Season amongst Study Participants in Rural Area (Table 10 and Fig. 10)

Among the study participants in rural area a negative correlation was observed between pH and gingival scores in winter season (–0.025) and a positive correlation was observed for the same in summer (+0.045), but this difference was found to be not statistically significant (>0.05).

### Correlation between pH and Gingival Scores according to Season amongst Study Participants in Urban Area (Table 11 and Fig. 11)

Among the study participants in urban area a positive correlation was observed between pH and gingival scores in winter season (+0.060) and a negative correlation was observed for the same in summer (–0.085), but observed values were found to be not statistically significant (>0.05).

**Table 9:** **Table 9: **Correlation between gingival index and pH according to season among study participants belonging to rural area

*Season*		*r-value*		*p-value*		*Significance*	
Winter		–0.025		>0.05		NS	
Summer		+0.045		>0.05		NS	

NS: Not significant

**Fig. 9:**
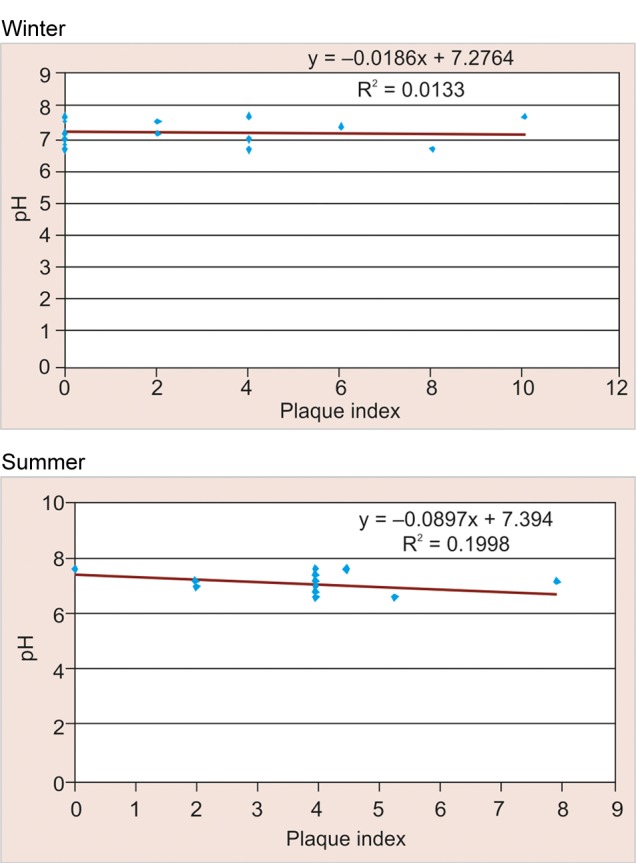
Correlation between pH and plaque scores according to season among study participants in urban area

## DISCUSSION

According to ayurveda with the changes in season, our body needs different foods to adjust itself to the outside environment.^16^ In Jaipur city, maximum average temperature in summers ranges between 33 and 45°C; whereas, minimum temperature ranges between 24 and 27°C. Similarly, in winter maximum average temperature ranges between 20 and 24°C, and minimum temperature ranges between 7 and10°C.^10^

Thus, due to contrast seasonal variation in the city, the food availability and consumption varies accordingly. Likewise in winter along with consumption of routine fruits and vegetables, sweets cuisines containing jaggary/gur are highly increased as they are believed to have warming properties. Similarly in summer season along with routine fruits and vegetables consumption of lassi (sweetened yoghurt) and chaach (buttermilk) are increased as these are believed to possess excellent coolant properties and also prevent oral dehydration. Sweets are an integral part of Rajasthani food which are not merely used as desserts but are consumed before and even along with the meal. Churma, gajak, ghevar are some of the popular sweets consumed in Rajasthan especially during winter season.^9^ As these sweets are solid and retentive in nature and thereby decreases the oral clearance time and possibly affecting the salivary parameters qualitatively and quantitatively.

Thus, the present study considers evidences about the seasonal variations (it is a component of time series which is defined as repetitive and predictable movement around the trend line in 1 year or less, which is detected by measuring the quantity of interest for small time intervals, such as days, weeks, months or quarters)^17^ in salivary parameters, and its possible effect upon plaque and gingival status amongst study population.

**Table 10:** **Table 10: **Correlation between plaque index and pH according to season among study participants belonging to urban area

*Season*		*r-value*		*p-value*		*Significance*	
Winter		– 0.115		>0.05		NS	
Summer		– 0.447		<0.05		Sig	

S: Significance; NS: Not significant

**Fig. 10:**
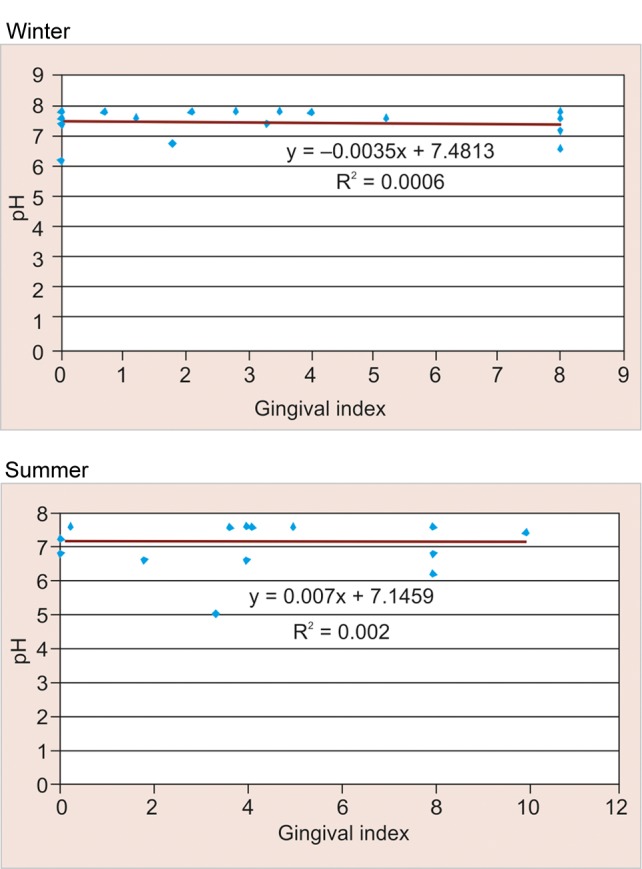
Correlation between pH and gingival scores according to season among study participants in rural area

**Table 11:** **Table 11**: Correlation between gingival index and pH according to season among study participants belonging to urban area

*Season*		*r-value*		*p-value*		*Significance*	
Winter		+ 0.060		>0.05		NS	
Summer		– 0.085		>0.05		NS	

NS: Not significant

**Fig. 11:**
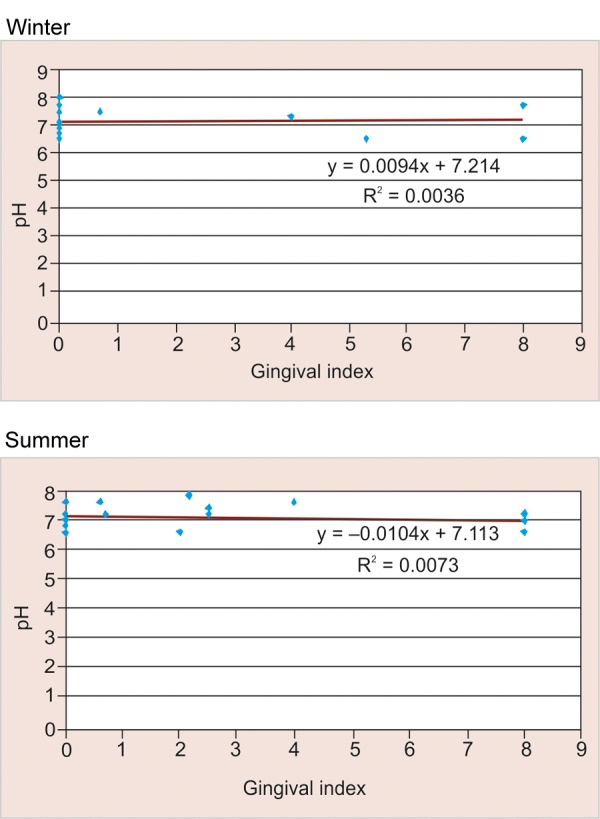
Correlation between pH and gingival scores according to season among study participants in urban area

### Comparison of Level of Hydration according to Season and Area

There was no difference in resting flow rate of saliva in both the seasons as unstimulated/resting flow rates are mainly affected due to the functioning of salivary glands; whereas, a comparative difference was observed in flow rates w.r.t area, i.e flow rates were higher among urban schoolchildren. This could be possible due to difference in food consumption patterns between the two areas. Findings were in contrast with following articles by MC Mazengo, E Soderling, P Alakuijala, J Tiekso, O Simell, Z Shi, N Lien, BN Kumar and G Holmboe- Ottesen in (2005)^18^ also reported minimal or no difference in food patterns among rural and urban Jiangsu schoolchildren and Anwar T Merchant, Mahshid Dehghan, Deanna Behnke Cook and Sonia S Anand in (2007)^19^ also suggested no differences in food consumption among rural and urban schoolchildren. H Hausen in (1994)^11^ in which no differences were found in mean salivary flow rates among rural and urban subjects.

### Comparison of Saliva Consistency according to Season and Area

Viscosity of saliva increases in winter as compared to summer among rural schoolchildren. This could be attributed due to increased sugar and snack consumption among rural schoolchildren particularly during winter. Observations were in line with a study by MC Mazengo, E Soderling, P Alakuijala, J Tiekso, O Simell, H Hausen in (1994),^11^ which state that saliva viscosity increases because there is increase in salivary protein concentration which was largely of plant origin as compared to urban diet which was animal protein dominated and MC Mazengo, E Soderling, P Alakujala, J Tiekso, J Tenovuo, O Simell, H Hausen in (1994)^11^ found that there was increase intakes of sucrose consumption in rural schoolchildren as compared to urban. These finding were in contrast to study conducted by Blay D, Astrom AN, Haugejorden O (2000)^20^, Nyandindi U, Palin Palokas T, Milen A, Robinson V, Kombe N in (1994)^21^where sugar and snack consumption was higher among urban schoolchildren; whereas, Z Shi, N Lien, BN Kumar and G Holmboe-Ottesen in (2005)^18^ reported no difference in sweet scores among rural and urban Jiangsu schoolchildren.

### Comparison of Saliva Quantity according to Season and Area

Saliva quantity was found to be very low in summer as compared to winter among rural schoolchildren. Reported findings were in contrast to an article by EM GhezzI, LA Lange and JA Ship (2000)^22^ in which no difference was observed in stimulated salivary flow rates in rural and urban study participants.

### Comparison of Buffering Capacity according to Season and Area

Salivary buffering capacity was found to be lower in winter season irrespective of the difference in area. This could be possible due to sweet cuisines containing jaggery/gur are increased in market and school canteens during winter season as they believed to have warming properties. Contrast findings were reported by MC Mazengo, E Soderling, P Alakuijala, J Tiekso, O Simell, H Hausen in (1994)^11^where salivary buffering capacity was found to be higher among rural subjects than urban as residents in rural area consume more fiber-rich diet.

### Mean pH Scores according to Season and Area

Scores were found to be higher in summer as compared to winter among both rural and urban schoolchildren, and this difference was statistically significant (<0.05) among participants belonging to rural area. Reason could be due to sweet cuisines containing jaggery/gur are increased in market and school canteens during winter and as these items are solid, sticky and more retentive in nature that adhere to the tooth surface for a longer duration thereby lowering down the pH particularly in winter season.

### Mean Plaque Scores (PI) according to Season and Area

Scores were found to be higher in winter as compared to summer among participants belonging to both the area. This could be attributed due to more consumption of sweet snacks which are solid, sticky and more retentive in nature that adhere to tooth surface for a longer duration and thereby decreasing the rate of oral clearance and thus increasing the plaque scores in winter.

### Mean Gingival Scores (GI) according to Season and Area

Scores were found to be higher in winter as compared to summer among participants belonging to both the area and this difference is found to be not statistically significant (> 0.05). Reason could be due to increased intake of retentive sweet snacks which adhere to tooth surface for a longer duration and thereby decreasing the rate of oral clearance and thus increasing the gingival scores in winter. Findings were in contrast to a study by MC Mazengo, E Soderling, P Alakuijala, J Tiekso, O Simell, H Hausen in (1994)^11^ in which gingival scores were higher among urban subjects as they consume more sucrose and less fiber-rich diet.

### Correlation between pH and Plaque Scores (PI) according to Season amongst Study Participants in Rural Area

In winter, positive correlation (+0.063) was observed between pH and plaque scores as compared to summer where negative correlation was observed for the same. This could be due to the reason that in winter season there is increased consumption of sweet snacks which are solid, sticky and retentive in nature which adhere to the tooth surface for a longer duration thus decreasing the oral clearance rate, lowering the salivary pH and thereby increasing the plaque scores.

### Correlation between pH and Plaque Scores (PI) according to Season amongst Study Participants in Urban Area

In winter, negative correlation (–0.447) was observed between pH and plaque scores which was found to be statistically significant (<0.05). This could be attributed due to improved oral hygiene practices among urban schoolchildren as compared to rural. Similar findings were reported by Blay D, Astrom AN, Haugejorden O (2000)^20^where increased oral hygiene practices among urban than rural residents.

### Correlation between pH and Gingival Scores (GI) according to Season amongst Study Participants in Rural Area

In winter, positive correlation (+0.045) was observed between pH and gingival scores as compared to summer where negative correlation was observed for the same. This could be due to the reason that in winter season there is increased consumption of sweet snacks which are solid, sticky and retentive in nature which adhere to the tooth surface for a longer duration thus decreasing the oral clearance rate lowering the salivary pH and thereby increasing the gingival scores.

### Correlation between pH and Gingival Scores according to Season amongst Study Participants in Urban Area

In winter, positive correlation (+0.060) was observed between pH and gingival scores; whereas in summer, negative correlation (–0.085) was observed for the same. This could be due to the reason that in winter season there is increased consumption of sweet snacks which adhere to the tooth surface for a longer duration thus decreasing the oral clearance rate, lowering the salivary pH and thereby increasing the gingival scores. Findings were in line to a study by MC Mazengo, E Soderling, P Alakuijala, J Tiekso, O Simell, H Hausen in (1994)^11^ in which gingival scores were higher among urban subjects as they consume more sucrose and less fiber-rich diet.

## SUMMARY AND CONCLUSION

- No difference in resting salivary flow rate was observed between the two seasons but was found to be comparatively higher among urban schoolchildren as compared to rural.
- Viscosity of saliva increases in winter as compared to summer among rural schoolchildren.
- Saliva quantity was found to be very low in summer as compared to winter among rural schoolchildren.
- Salivary buffering capacity was found to be lower in winter season irrespective of the difference in area.
- Salivary pH scores were found to be higher in summer as compared to winter among both rural and urban schoolchildren, and this difference was statistically significant (<0.05) among participants belonging to rural area.
- Mean plaque scores were found to be higher in winter as compared to summer among participants belonging to both the area but the observed difference was found to be not statistically significant (>0.05).
- Mean gingival scores were found to be higher in winter as compared to summer among participants belonging to both the area and this difference is found to be not statistically significant (>0.05).
- Positive correlation (+ 0.063) was observed between pH and plaque scores in winter among study participants of rural area; whereas, negative correlation (–0.447) was observed in winter among study participants of urban area which was found to be statistically significant (<0.05).
- Positive correlation (+ 0.045) was observed between pH and gingival scores in winter among study participants of rural area; whereas, positive correlation (+0.060) was observed in winter among study participants of urban area which was found to be not statistically significant (>0.05).

To conclude, it can be said that there is a need for dietary counseling and basic oral health care in the study area irrespective of the season. Food consumption patterns differ significantly in winter as compared to summer thereby affecting the oral clearance rate which directly or indirectly affects unstimulated and stimulated salivary patterns and plaque and gingival status.
